# Matched Stochastic Resonance Enhanced Underwater Passive Sonar Detection under Non-Gaussian Impulsive Background Noise

**DOI:** 10.3390/s24092943

**Published:** 2024-05-06

**Authors:** Haitao Dong, Shilei Ma, Jian Suo, Zhigang Zhu

**Affiliations:** 1 Xi’an Key Laboratory of Intelligent Spectrum Sensing and Information Fusion, Xidian University, Xi’an 710071, China; donghaitao@xidian.edu.cn; 2 Shaanxi Union Research Center of University and Enterprise for Intelligent Spectrum Sensing and Information Fusion, Xidian University, Xi’an 710071, China; 3 School of Marine Science and Technology, Northwestern Polytechnical University, Xi’an 710072, China; slma@mail.nwpu.edu.cn (S.M.); jiansuo@mail.nwpu.edu.cn (J.S.)

**Keywords:** matched stochastic resonance (MSR), passive sonar detection, weak signal detection, non-Gaussian impulsive noise

## Abstract

Remote passive sonar detection with low-frequency band spectral lines has attracted much attention, while complex low-frequency non-Gaussian impulsive noisy environments would strongly affect the detection performance. This is a challenging problem in weak signal detection, especially for the high false alarm rate caused by heavy-tailed impulsive noise. In this paper, a novel matched stochastic resonance (MSR)-based weak signal detection model is established, and two MSR-based detectors named MSR-PED and MSR-PSNR are proposed based on a theoretical analysis of the MSR output response. Comprehensive detection performance analyses in both Gasussian and non-Gaussian impulsive noise conditions are presented, which revealed the superior performance of our proposed detector under non-Gasussian impulsive noise. Numerical analysis and application verification have revealed the superior detection performance with the proposed MSR-PSNR detector compared with energy-based detection methods, which can break through the high false alarm rate problem caused by heavy-tailed impulsive noise. For a typical non-Gasussian impulsive noise assumption with α=1.5, the proposed MSR-PED and MSR-PSNR can achieve approximately 16 dB and 22 dB improvements, respectively, in the detection performance compared to the classical PED method. For stronger, non-Gaussian impulsive noise conditions corresponding to α=1, the improvement in detection performance can be more significant. Our proposed MSR-PSNR methods can overcome the challenging problem of a high false alarm rate caused by heavy-tailed impulsive noise. This work can lay a solid foundation for breaking through the challenges of underwater passive sonar detection under non-Gaussian impulsive background noise, and can provide important guidance for future research work.

## 1. Introduction

Weak signal detection is a long-term, important technology for underwater passive sonar systems for port and coastal security [1,2,3,4]. Through experiments and modeling, an excellent description of ship-radiated noise as a combination of broadband noise and line spectral signatures has been provided [5,6,7]. These line spectral signatures, related to the rotation of engines, shaft-line dynamics, and propeller cavitations, are mostly adopted by various algorithms for passive sonar detection, tracking, and classification. In recent years, the development trend of low-frequency sonar is becoming increasingly apparent. Theoretical and experimental results have proven that there are abundant low-frequency periodic components that are related to the engine and propeller. Therefore, remote passive sonar detection with very-low-frequency (f≤100 Hz) signatures has attracts lots of attention [8,9,10,11].

**Figure 1:**
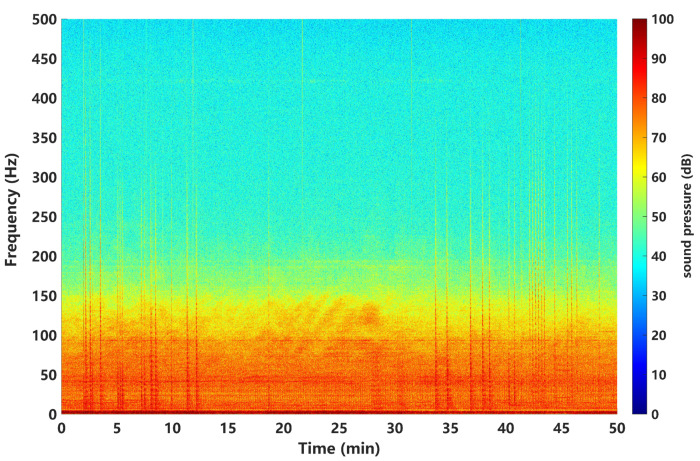
Time–frequency map of typical ocean ambient noise measured in the South China Sea (50 min).

A variety of detection approaches to weak spectral lines have been proposed, such as statistical energy detectors [12,13], adaptive line enhancers [14,15], and neural networks [16,17]. Most studies adopted the Gaussian assumption to describe the marine ambient noise in theoretical applications due to its simplicity. However, this is not always appropriate to match real noisy processes, as it can lead to a worse detection performance in practice. Much of the experimental and modeling research has shown that the underwater acoustic noise environment is non-Gaussian and impulsive in nature [18,19,20,21]. In fact, the source distribution range and quantity of low-frequency marine environmental noise fields is complex, including industrial noise, distant ship-radiated noise, broadband explosion noise, and marine biological noise. In the past 30 years, more and more researchers addressed the issue of complex marine noise analyses and modelings. Typical modeling methods include Gaussian Mixture (GM) [22], Generalized Gaussian Distribution (GG) [12], α stable distribution model (SαS) [23], and Generalized Autoregressive Conditional Heteroscedasticity (GARCH) [24]. The SαS model is a generalized noise distribution model that has good adaptability to spike state noise with significant pulses. It has been applied by many scholars in the statistical modeling of complex marine environmental noise, especially in the very-low-frequency band of marine environmental noise. Figure 1 shows a set of 50-min time–frequency maps of typical measured marine ambient noise in the South China Sea. It can be seen that there is a large amount of strong random impulsive interference, mainly energy distributed in the lower frequency, below 150 Hz. Therefore, in the scenario of very-low-frequency remote passive detection, the detection problem is challenging and should not simply assume an ideal Gaussian noise environment.

As is known for non-Gaussian signal detection, an optimal receiver for signal detection under non-Gaussian noise is made by constructing nonlinear filters to approximate the optimal nonlinear transfer function [25]. Based on this, nonlinear preprocessing methods were generally adopted, such as soft limiters [26] and infinite filter banks [27]. These indicate that detection theories and methods under non-Gaussian noise should be nonlinear. However, the optimized nonlinear systems often have complex structures. Stochastic resonance (SR) is a nonlinear phenomenon that has the distinct merit of enhancing the signal energy by exploiting the noise energy, and therefore increasing the signal-to-noise ratio (SNR). Studies have shown that using SR in weak signal detection is effective, especially under low-SNR conditions [28,29,30,31,32]. From the comprehensive perspective of performance and cost, the suboptimal stochastic resonance method has more potential for application due to its simple and efficient design [33,34,35,36], and by utilizing the SR, the proposed method can achieve a better performance in comparison to matched filter [37,38]. Nevertheless, these work mainly realized SR by adding an appropriate noise level to maximize the output SNR. Adding appropriate noise with a low noise intensity might be a suitable option, but this has restrictions regarding how to remove a high level of noise, especially in a low-SNR region. Therefore, rather than adjusting the input noise level, tuning the signal structure and (or) system parameters might be more suitable for practical signal processing applications. Xu et al. [39] extended the concept of SR and proposed a parameter-induced stochastic resonance (PSR) by tuning system parameters, which greatly promoted the development of stochastic resonance for practical applications. Theoretical analyses have shown that the optimal SR effect will always be obtained by only changing the system parameters, which indicates the superiority and feasibility of parameter tuning [13,40]. This can be regarded as a special nonlinear filter, which has the distinct advantage of enhancing weak signals compared to traditional filters [41,42,43]. To date, there have been many theoretical investigations into non-Gaussian-noise-influenced SR phenomena, which have shown the effectiveness of SR in dealing with non-Gaussian and impulsive noise [44,45,46,47]. These indicate the potential of SR in breaking through the challenge of weak signal detection under non-Gaussian noise.

Motivated by the aforementioned analyses, a matched stochastic resonance (MSR) method is proposed to address the challenging problem of underwater passive sonar detection under non-Gaussian impulsive background noise. Focusing on this problem, a brief conference report on an MSR-based weak signal detection scheme was investigated in the previous study, which preliminarily demonstrated the effectiveness of SR for ship-radiated line spectrals detection under heavy-tailed Lévy background noise [48]. However, it was simply realized, and was not sufficient in theory. In view of this, in this paper, MSR-based weak signal detectors named MSR-PED and MSR-PSNR are proposed on the basis of a theoretical analysis of the matched-output response. Numerical analyses and application verifications have revealed that the proposed methods are superior, especially for the proposed MSR-PSNR under low-false-alarm-rate conditions. The main contributions of this work can be summarized as follows:

(1) A novel MSR-based weak signal detection model is established, and an optimized MSR-PSNR detector is proposed on the basis of a theoretical analysis of MSR output response.

(2) Comprehensive detection performance analyses of both Gasussian and non-Gasussian impulsive noise conditions are conducted, which revealed the superior performance of the proposed detector under non-Gasussian impulsive noise.

(3) A superior detection performance can be achieved with the proposed MSR-PSNR detector in comparison with energy-based detection methods, which can break through the high false alarm rate problem caused by heavy-tailed impulsive noise.

(4) This work can lay a solid foundation for breaking through the challenges and problems faced in underwater passive sonar detection under a low SNR and complex non-Gaussian and impulsive ambient noise, and can provide important guidance for future research work.

The rest of the paper is organized as follows. In Section 2, the signal model for passive sonar detection is presented with the non-Gaussian impulsive noise assumption. In Section 3, the weak signal detectors using the matched stochastic resonance (MSR) method were proposed. The simulation outcomes of both Gasussian and non-Gasussian impulsive noise conditions are evaluated in Section 4, and experimental verification is further given in Section 5. Finally, concluding remarks are drawn in Section 6.

## 2. Signal Model

### 2.1. Periodogram-Based Energy Detector (PED) for Passive Sonars

Ship-radiated noise can generally be modeled as a combination of broadband noise and sinusoidal tonal signals, of which the sinusoidal tonal signals, the so-called line spectrums, have the merits of high stability and intensity, meaning that they are widely used for target detection, orientation, location, tracking, and recognition. The line spectral signatures could be adopted as the periodical signals. In the actual work process, the received ship-radiated signal can be written as follows:

(1) $$\begin{array}{cc} r(t) & =s(t)+n(t)\\ \; & =\sum_{i=1}^{M}A_{i}cos\left(2\pi f_{i}t+\varphi_{i}\right)+n\left(t\right) \end{array}$$

where *M* is the number of line spectral signatures; $f_{i}$ represents the line spectral frequencies; $A_{i}$ and $\varphi_{i}$ are the corresponding amplitudes and phases, respectively. n(t) represents the combination of radiated broadband noise and ocean ambient noise.

Usually, the detection problem can be regarded as a binary hypothesis testing problem, as follows:

(2) $$\begin{array}{c} \left\{\begin{array}{c} H_{0}:r\left(t\right)=n\left(t\right)\;\\ H_{1}:r\left(t\right)=s\left(t\right)+n\left(t\right) \end{array}\right. \end{array}$$

where $H_{0}$ is the null hypothesis that refers to noise without signal, and $H_{1}$ is the alternative hypothesis referring to the periodic signal and additive noise.

According to the Parseval theorem:

(3) $$\sum_{l=0}^{N-1}|r\left[l\right]{|}^{2}=\frac{1}{N}\sum_{k=0}^{N-1}|Y\left[k\right]{|}^{2}$$

where $r\left[l\right]=r\left(lT_{s}\right)$ is the sample of r(t), *N* is the sample number, and Y[k] is the Fourier transform of sample sequence r[l], which can also be represented as follows:

(4) $$Y\left[k\right]=Y\left[f\right]\delta \left(f-\frac{k}{N}f_{s}\right)={Y\left[f\right]|}_{f=\frac{k}{N}f_{s}}$$

in which $f_{s}=1/T_{s}$ is the sampling frequency and Y[f] is the discrete Fourier transformation (DFT) of r(t). Based on the definition of the periodogram:

(5) $$\hat{P}\left[k\right]=\frac{1}{N}|\sum_{n=0}^{N-1}r\left[l\right]exp\left(-2\pi jkn/N\right){|}^{2}=\frac{1}{N}|Y{\left[f\right]}_{f=\frac{k}{N}f_{s}}{|}^{2}$$

Then, the test statistic can be given as follows:

(6) $$T\left(r\right)=\sum_{k=0}^{N-1}\hat{P}\left[k\right]\overset{H_{1}}{\underset{H_{0}}{⋛}}\;\gamma_{PED}$$

in which $\gamma_{\mathrm{PED}}$ is the decision threshold that is chosen to satisfy $P_{FA}=\alpha$ under the Neyman–Pearson (N-P) criterion [49].

### 2.2. Non-Gaussian Impulsive Noise Assumption

For weak signal detection, in theoretical applications, the Gaussian assumption is widely used to describe background noise for simplicity in, while this not always appropriate to describe real noisy processes. As mentioned previously, the VLF ocean ambient noise is non-Gaussian and impulsive in nature. The stable Lévy distribution provides an excellent way to model complicated noise process, and was therefore employed and analyzed in this paper.

**Figure 2:**
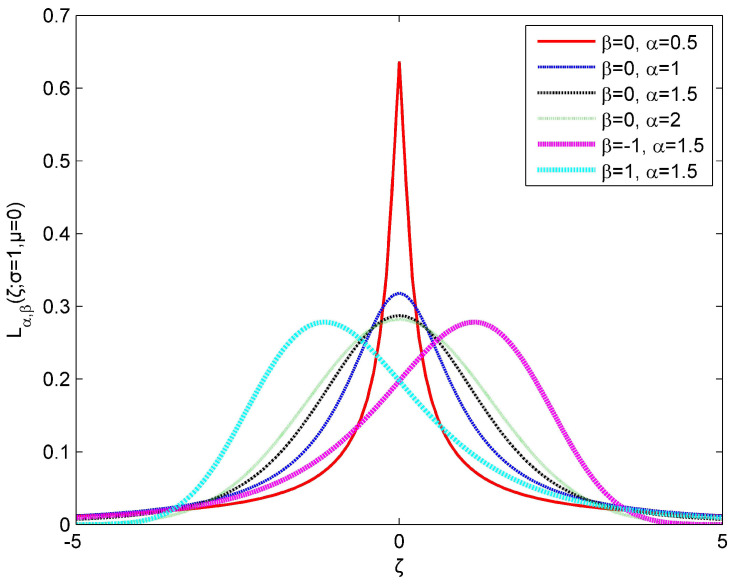
Probability density functions for Lévy distribution $L_{\alpha ,\beta }\left(\zeta ;\sigma ,\mu \right)$ with different stability indexes and asymmetry parameters.

Assume that ζ(t) obeys Lévy distribution $L_{\alpha ,\beta }$(ζ;σ,μ); its characteristic function can be given as follows [50]:

(7) $$\Phi \left(k\right)\;=\;\;\left\{\begin{array}{c} \exp\left\{\;-\;\sigma^{\alpha }{\left|k\right|}^{\alpha }[1\;-\;i\beta sgn\left(k\right)\tan(\frac{\alpha \pi }{2})]\;+\;ik\mu \right\}\;\alpha \neq 1\\ \exp\left\{-\;\sigma^{\alpha }|k|[1\;-\;i\beta \frac{\pi }{2}sgn\left(k\right)\log_{10}(|k|)]\;+\;ik\mu \right\}\;\alpha =1 \end{array}\right.$$

where α∈(0,2] denotes the stability index that describes an asymptotic power law of the Lévy distribution: the smaller the α, the stronger the impulsive characteristics. β∈[−1,1] is the asymmetry parameter, which represents the distributed left deviation, right deviation, and symmetry for positive, negative, and zero, respectively. Moreover, μ∈R is the mean parameter that represents the center of distribution; η∈(0,+∞) is the scale parameter used to measure the degree of deviation from the mean. α=2 represents the Gaussian distribution and α=1 represents the well-known Cauchy distribution. The probability density functions for Lévy distribution $L_{\alpha ,\beta }\left(\zeta ;\sigma ,\mu \right)$ with different stability indexes and asymmetry parameters are shown in Figure 2, which can be considered a generalized noise model for real ocean ambient noise. In this paper, the Janicki–Weron algorithm is employed to generate the Lévy distribution sequence [48]. Since, for α<2, there is no finite variance in thhe noise, the noise power $S_{0}$ can be obtained by the following:

(8) $$S_{0}={\left(C_{g}\eta \right)}^{1/\alpha }/C_{g}$$

where $C_{g}\approx 1.78$ is the exponential form of Euler’s constant. The noise intensity $\hat{D}$ can be estimated as $\hat{D}={\left(\eta \right)}^{\alpha }$. To ensure that the definition of SNR is in accordance with the white Gaussian noise, the input SNR can be expressed by normalizing $2C_{g}$, as presented below:

(9) $$\;{\mathrm{SNR}}_{in}=10\log_{10}\frac{1}{2C_{g}}{\left(\frac{\sqrt{E}}{S_{0}}\right)}^{2}$$

in which *E* represents the signal energy.

## 3. Matched Stochastic Resonance-Based Weak Signal Detector

### 3.1. Classical Bistable Stochastic Resonance (CBSR)

Stochastic resonance is a nonlinear phenomenon, of which the most significant characteristic is that a certain relationship between signal, noise, and system parameter can cause a transfer of energy from a random process (noise) to a periodic process (signal). Such a phenomenon is commonly described by the Brownian motion:

(10) $$m\ddot{x}+\gamma \dot{x}=-\dot{V}\left(x\right)+s\left(t\right)+n\left(t\right)$$

in which *m* is the mass of the Brownian particle, and *x* is the displacement trajectory of the Brownian particle; γ is the coefficient of friction. $s\left(t\right)=A_{0}cos\left(\omega_{0}t+\varphi_{0}\right)$ represents a periodic signal with $A_{0}$, $\omega_{0}$, and $\varphi_{0}$ as the amplitude, driving frequency, and the initial phase, respectively. $n\left(t\right)=\sqrt{2D}\eta \left(t\right)$ represents the noise item, where *D* is the noise intensity and η(t) represents an additive noise. V(x) is the quartic double well (QDW) potential function, as presented below:

(11) $$V\left(x\right)=-\frac{1}{2}ax^{2}+\frac{1}{4}bx^{4}$$

where *a* and *b* denote the barrier potential parameters with a positive real value. One maximum and two minimum values of potential V(x) can be found at $x_{0}=0$ and $\pm x_{m}=\pm \sqrt{a/b}$, respectively, and the difference between the maximum and minimum represents the threshold of the system or the energy barrier with the amplitude $\Delta V=a^{2}/4b$.

For a deterministic, static, nonlinear system with fixed potential parameters, there are two situations in which it is driven by external forces:

(1) In the situation of a nonlinear system that is only subjected to a stochastic forcing signal (referred to as the $H_{0}$ assumption), the statistical properties of noise-induced particle transition between two potential wells can be characterized by the famous Kramers rate as follows:

(12) $$r_{K}=\frac{\omega_{0}\omega_{b}}{\sqrt{2}\pi \gamma }\exp\left(-\frac{\Delta V}{D}\right)$$

where $\omega_{0}={\left[V^{\prime \prime }\left(\pm x_{m}\right)\right]}^{1/2}={\left(2a\right)}^{1/2}$ and $\omega_{b}={\left[V^{\prime \prime }\left(x_{0}\right)\right]}^{1/2}={\left(a\right)}^{1/2}$ represent the characteristic frequencies of the system with the potential minimum and maximum value.

(2) When the system is simultaneously excited by noise and weak external periodic signals (refer to the assumption of $H_{1}$), the bistable potential will be periodically modulated, and the left and right potential wells will alternately rise or decrease over the period of $T=1/f_{0}$. When the amplitude of the weak periodic signal $A_{0}$ is greater than the system critical threshold $A_{c}$ ($A_{c}=\sqrt{4a^{3}/\left(27b\right)}$), with the assistance of a certain amount of noise energy, even in the situation of $A_{1}\ll A_{c}$, it can induce a periodic well-to-well particle transition, triggering a synergistic matching mechanism between the nonlinear system, noise, and external periodic excitation. At this time, the Brownian particle undergoes periodic back and forth transitions, resulting in a modified form of the Kramers rate $r_{K}^{\prime }$, as follows:

(13) $$r_{K}^{\prime }=\frac{\omega_{0}\omega_{b}}{2\pi \gamma }\exp\left(-\frac{\Delta V\pm A_{0}\cos\left(\omega_{0}t+\varphi_{0}\right)}{D}\right)$$

where the frequency of the periodic signal input to the system is much smaller than the rate at which the system tends to equilibrium at each potential well: $\omega_{0}\ll 2a$.

To better characterize the performance of SR, the power spectra G(ω) of the system response is usually analyzed via SNRI measurements. For the nonlinear SR system, the output power spectra can be expressed as follows [51]:

(14) $$G\left(\omega \right)=G_{s}\left(\omega \right)+G_{n}\left(\omega \right)$$

in which $G_{s}\left(\omega \right)$ and $G_{n}\left(\omega \right)$ represent the output power spectra of signal and noise, respectively:

(15) $$G_{s}\left(\omega \right)=\frac{\pi }{2}{\left(\frac{Ax_{m}}{D}\right)}^{2}\frac{4r_{K}^{2}}{4r_{K}^{2}+\omega_{0}^{2}}\left[\delta \left(\omega -\omega_{0}\right)+\delta \left(\omega +\omega_{0}\right)\right]$$

and

(16) $$G_{n}\left(\omega \right)=\left[1-\frac{1}{2}{\left(\frac{Ax_{m}}{D}\right)}^{2}\frac{4r_{K}^{2}}{4r_{K}^{2}+\omega_{0}^{2}}\right]\frac{4r_{K}x_{m}^{2}}{4r_{K}^{2}+\omega_{0}^{2}}$$

where $\left[1-\frac{1}{2}{\left(\frac{A_{0}x_{m}}{D}\right)}^{2}\frac{4r_{K}^{2}}{4r_{K}^{2}+\Omega^{2}}\right]$ represents the correction coefficients for the noise power spectrum. Under the assumption of adiabatic approximation, the following could also be assumed:

(1) When the amplitude of the input periodic signal is small enough or there is no periodic signal (refer to the $H_{0}$ assumption), the value of the correction coefficient approaches 1. At this time, noise has no effect on the output signal, and SR can not occur. The frequency response of the system output follows a Lorentz distribution.

(2) As the amplitude of the periodic signal gradually increases (refer to $H_{1}$ assumption), the correction coefficient gradually decreases, and the energy transferred from noise to the signal gradually increases. According to the characteristics of the Lorentz distribution, the energy transfer from noise to the signal tends to be at its maximum when the following the time-scale matching conditions for generating the random resonance effect are met [28].

(17) $$r_{K}=\frac{\omega_{0}}{\pi }$$

where $\omega_{0}=2\pi f_{0}$.

It can be seen that the Kramers rate $r_{K}$ is related to the output power spectra G(ω), as well as the system’s potential parameters, noise intensity *D*, and driving frequency $f_{0}$ described in Equation (13). Through combining Equations (15) and (16), the output SNR can finally be obtained and approximated as follows [51]:

(18) $$\begin{array}{cc} {\mathrm{SNR}}_{\mathrm{output}} & =\frac{\pi }{2}{\left(\frac{Ax_{m}}{D}\right)}^{2}r_{K}\times {\left[1-\frac{1}{2}{\left(\frac{Ax_{m}}{D}\right)}^{2}\frac{4{r_{k}}^{2}}{4{r_{k}}^{2}+2\pi f_{0}^{2}}\right]}^{-1}\\ & \approx \sqrt{2}\Delta V{\left(\frac{A}{D}\right)}^{2}\exp^{-\Delta V/D} \end{array}$$

Consequently, the SNRI of the bistable SR system can be obtained in theory:

(19) $$\mathrm{SNRI}=\frac{{\mathrm{SNR}}_{\mathrm{output}}}{{\mathrm{SNR}}_{\mathrm{input}}}\approx \frac{4\sqrt{2}\Delta V}{D}\exp^{-\Delta V/D}$$

in which the output performance is determined by the noise intensity *D*, the damping factor γ, and the barrier height of the nonlinear system ΔV.

### 3.2. Framework of Matched Stochastic Resonance (MSR)

The definition of matched stochastic resonance can be described as follows: for a dynamic nonlinear system, under the constraint of the stochastic resonance effect, the nonlinearity of the system can be parameterized and optimized to achieve the matched output and maximize the signal-to-noise ratio improvement (SNRI). Its generalized mathematical represention can be presented as follows: follows [13,31,32],

(20) $$\begin{array}{c} \begin{array}{cc} \max_{a,b,\gamma ,...} & \mathrm{SNRI}\\ s.t. & \mathrm{SR}\;\mathrm{conditions} \end{array} \end{array}$$

where SR conditions can be obtained by classical theories and methods, including time-scale matching conditions [28,39], stability conditions [52,53], threshold conditions [54], amplitude gain under weak noise limit conditions [55], etc.

Assume a periodic forcing signal with an amplitude *A* and a frequency $f_{0}$ is applied to the particle with an overdamped first-order bistable QDW potential. A matched relationship between the signal frequency, noise intensity, and nonlinear system parameters can be explored by maximizing system SNRI under the constraints of the SR conditions. Since *A* and *D* of the input noisy signal are generally deterministic in practice, maximizing system SNRI requires that the noise is matched with system parameters. This can be formed using the optimization problem presented below:

(21) $$\begin{array}{c} \begin{array}{cc} \max_{a,b} & \mathrm{SNRI}\\ s.t. & r_{K}=2f_{0}\\ \; & A<A_{c}<\sqrt{2D}\\ \; & \mathrm{SNRI}>1 \end{array} \end{array}$$

Note that the time-scale matching condition is generalized in terms of statistic meaning, and the constraint of SNRI>1 is to ensure the output SNR is increased using the SR approach. Then, the optimization problem in Equation (21) can be rewritten with respect to ΔV, as follows:

$$\Delta V_{opt}=\underset{\Delta V}{\arg\max}\;\;\mathrm{SNRI}$$

The mathematical matched potential parameters’ relationship can be obtained to the satisfaction of the SR matching principle, as presented below:

(22) $$\left\{\begin{array}{c} a_{opt}=2\sqrt{2}\pi {\hat{f}}_{0}e\\ b_{opt}=a_{opt}^{2}/\left(4\hat{D}\right) \end{array}\right.$$

where ${\hat{f}}_{0}$ and $\hat{D}$ represent the estimation of the signal frequency $f_{0}$ and the noise intensity *D*, respectively. e is the natural logarithm.

Consequently, the ${\mathrm{SNR}}_{\mathrm{output}}$ in Equation (18) can be rewritten, as presented below:

(23) $${\mathrm{SNR}}_{\mathrm{output}}=\frac{16A_{0}^{2}}{a_{\mathrm{opt}\;}^{2}\left(2+\pi^{2}/2\right)}/\left(\frac{a_{\mathrm{opt}\;}}{b_{\mathrm{opt}\;}}-\frac{16A_{0}^{2}}{a_{\mathrm{opt}\;}^{2}\left(2+\pi^{2}/2\right)}\right)$$

Due to the constraint of SNRI>1, the following relationship should be satisfied:

(24) $$\left\{\begin{array}{c} \frac{a_{opt}}{b_{opt}}-\frac{16A^{2}}{a_{opt}^{2}\left(2+\pi^{2}/2\right)}>0\\ \frac{16}{b_{opt}\left(2+\pi^{2}/2\right)}>\frac{a_{opt}}{b_{opt}}-\frac{16A^{2}}{a_{opt}^{2}\left(2+\pi^{2}/2\right)} \end{array}\right.$$

This can be further simplified under the assumption of adiabatic approximation as follows:

(25) $$\left\{\begin{array}{c} 0<A<\frac{a_{opt}}{4}\sqrt{\frac{a_{opt}\left(2+\pi^{2}/2\right)}{b_{opt}}}\\ a_{opt}\ll 2\sqrt{2}\pi e \end{array}\right.$$

Consequently, the optimization problem in Equation (21) can be simplified, as presented below:

(26) $$\begin{array}{c} \begin{array}{ccc} \; & \max_{a,b} & \mathrm{SNRI}\\ \; & s.t. & a=2\sqrt{2}\pi {\hat{f}}_{0}e\\ \; & \; & b=a^{2}/\left(4\hat{D}\right)\\ \; & \; & 0<A<\frac{a}{4}\sqrt{\frac{a\left(2+\pi^{2}/2\right)}{b}}\\ \; & \; & a\ll 2\sqrt{2}\pi e \end{array} \end{array}$$

**Figure 3:**
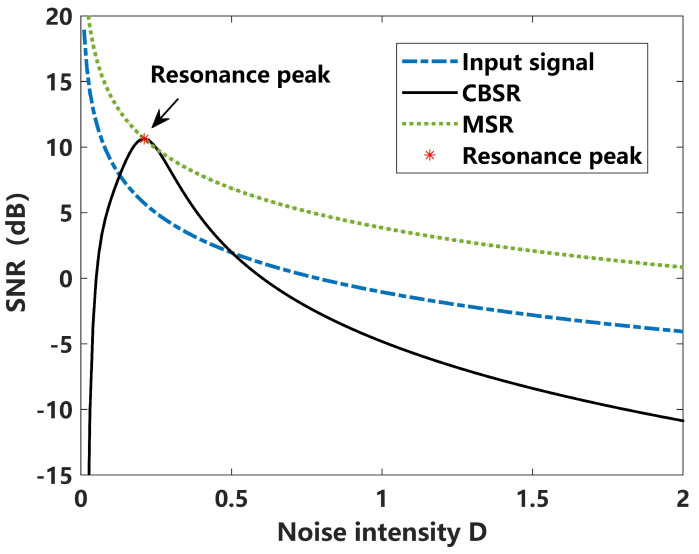
A comparison MSR outputting SNR responses under different noise intensities *D*.

In this way, the weak signal that is hidden against the heavy background can be enhanced and detected. A comparison of output SNR curves corresponding to different input noise intensities is given in Figure 3. For a deterministic, static, nonlinear system of CBSR with fixed system parameters, the curve of the output SNR varied with noise intensity *D*, forming a “resonance” curve, where there exists an optimal noise intensity value $D_{opt}$ that can achieve the maximum ${\mathrm{SNR}}_{\mathrm{output}}$. For the curve corresponding to the MSR, it can be seen that the system parameters can be optimized under any conditions.

According to the adiabatic approximation theorem, the MSR system is restricted by the input signal with a low frequency $f_{0}$, where $f_{0}\ll 1$ Hz. For practical, large input signal frequencies with tens to thousands of hertz, a frequency-rescaling preprocessing technique can be utilized to satisfy the assumption. By introducing a scaling factor α in the process of solving the Runge–Kutta algorithm, the signal frequency $f_{c}$ is equivalently converted to a desirable value $f_{0}=f_{c}/\alpha$, as presented below:

(27) $$s\left[k\right]=s\left(kT_{s}\right)=A_{c}\cos\left(2\pi \frac{f_{c}}{\alpha }\left(k\alpha T_{s}\right)\right)=A_{c}\cos\left(2\pi f_{0}k\alpha T_{s}\right)$$

in which $T_{s}$ is the sampling time. Generally, the $f_{0}$ should be small enough, and in this paper the empirical value is set to $f_{0}=0.005$ Hz.

### 3.3. MSR-Based Passive Sonar Detection

**Figure 4:**
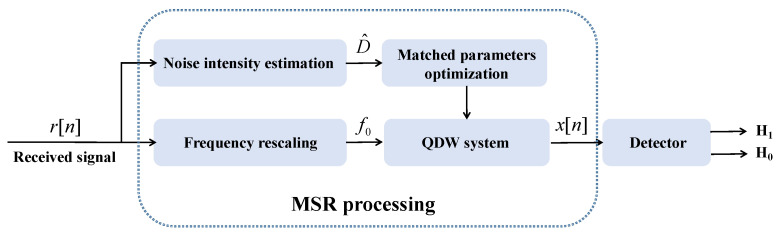
The framework of the proposed MSR-based passive sonar detection.

The MSR-based detection framwork is illustrated in Figure 4. The matched parameters’ optimization is carried out according to Equation (26), and computed to obtain x[n] with the classical Runge–Kutta algorithm. For the MSR-based detector, Equation (2) can be rewritten as follows:

(28) $$\begin{array}{c} \left\{\begin{array}{c} H_{0}:x\left(t\right)=f\left(n\left(t\right)\right)\;\\ H_{1}:x\left(t\right)=f\left(s\left(t\right)+n\left(t\right)\right) \end{array}\right. \end{array}$$

where f(·) is a nonlinear function that represents the SR effects. According to the output characteristics of the MSR output, two detection methods are provided.

#### 3.3.1. Periodogram-Based Energy Detector (MSR-PED)

Generally, a periodogram-based energy detector subjected to the frequency domain is superior to broadband energy detection methods in weak signal detection problems. In this way, the test statistics of MSR-based PED can be given as follows:

(29) $$T\left(x\right)=\frac{1}{2M}\sum_{k=k_{0}-M}^{k_{0}+M}|X\left[k\right]{|}^{2}\overset{H_{1}}{\underset{H_{0}}{⋛}}\;\gamma_{MSR-PED}$$

where X[k] is the discrete Fourier transform (DFT) to the MSR output x[n], $k_{0}$ represents the location corresponding to the signal frequency $f_{0}$, 2M is the length number to bandpass filter, and $\gamma_{\mathrm{MSR}-\mathrm{PED}}$ is the decision threshold that is chosen to satisfy $P_{FA}=\alpha$ under the Neyman–Pearson criterion.

#### 3.3.2. Peak SNR-Based Detector (MSR-PSNR)

According to the MSR optimization model in Equation (26), the MSR effect is achieved with a maximized SNRI index. From the perspective of designing the test statistics, utilizing the output SNR should be an intuitively better choice. In this way, a peak SNR-based detector is designed with the MSR output, as presented below:

(30) $$T\left(x\right)=10\log_{10}\left(\frac{N_{0}*X\left[k_{0}\right]}{\sum_{k=1}^{N_{0}/2}X\left[k\right]-X\left[k_{0}\right]}\right)\overset{H_{1}}{\underset{H_{0}}{⋛}}\;\gamma_{MSR-PSNR}$$

where $\gamma_{\mathrm{MSR}-\mathrm{PSNR}}$ is the corresponding decision threshold.

## 4. Numerical Analyses

To evaluate and verify the detection performance of the proposed methods, numerical analyses are conducted in this section. According to the non-Gaussian impulsive noise assumption given in Section 2, three typical types of noise—α=2, α=1.5, and α=1—are adopted to reveal the performance under different Gaussian and non-Gaussian impulsive conditions. As is known, the smaller the stability index α, the stronger the impulsive characteristics. α=2 refers to the Gaussian noise, and α=1.5 and α=1 refer to different degrees of impulsive noise. Note that in the real ocean ambient environment, the stability index is generally larger than α≥1.5, and in the case of an artificial interference environment with air guns, the impulsive noise background can be modeled by α=1.

A ship-radiated line spectral signature is simulated with a single 10 Hz periodic signal for simplicity. The simulation parameters are given as follows to better reveal its output performance: signal frequency $f_{0}=10$ Hz, Gaussian noise variance $\sigma_{n}^{2}=1$, sampling frequency $f_{s}=2$ kHz, and discrete signal sampling points N=6000. For the low reference frequency of $f_{0}=0.005$ Hz, according to Equation (26), $a_{opt}=0.12$, $b_{opt}=0.0072$, and the scale transformation factor is α=2000. In view of the theoretical analysis of the signal-to-noise ratio gain of a bistable system, it can be concluded that when the amplitude of the input periodic signal satisfies A<0.4217, the SNRI of the MSR system can be greater than 1. Therefore, a normalization preprocessing is performed of the received signal r[n].

Generally, prior knowledge of passive sonar detection is lacking, so the matched filtering (MF) method cannot be realized. However, this is known as the optimal detector under Gaussian noise and is the upper bound of energy-based detectors. In the following analyses, the MF and MSR-MF are utilized, aiming to provide a comprehensive understanding of the proposed MSR-PED and MSR-PSNR detection methods in both Gaussian and non-Gaussian conditions.

### 4.1. Detection Performance Analysis under Gaussian Noise (α=2)

**Figure 5:**
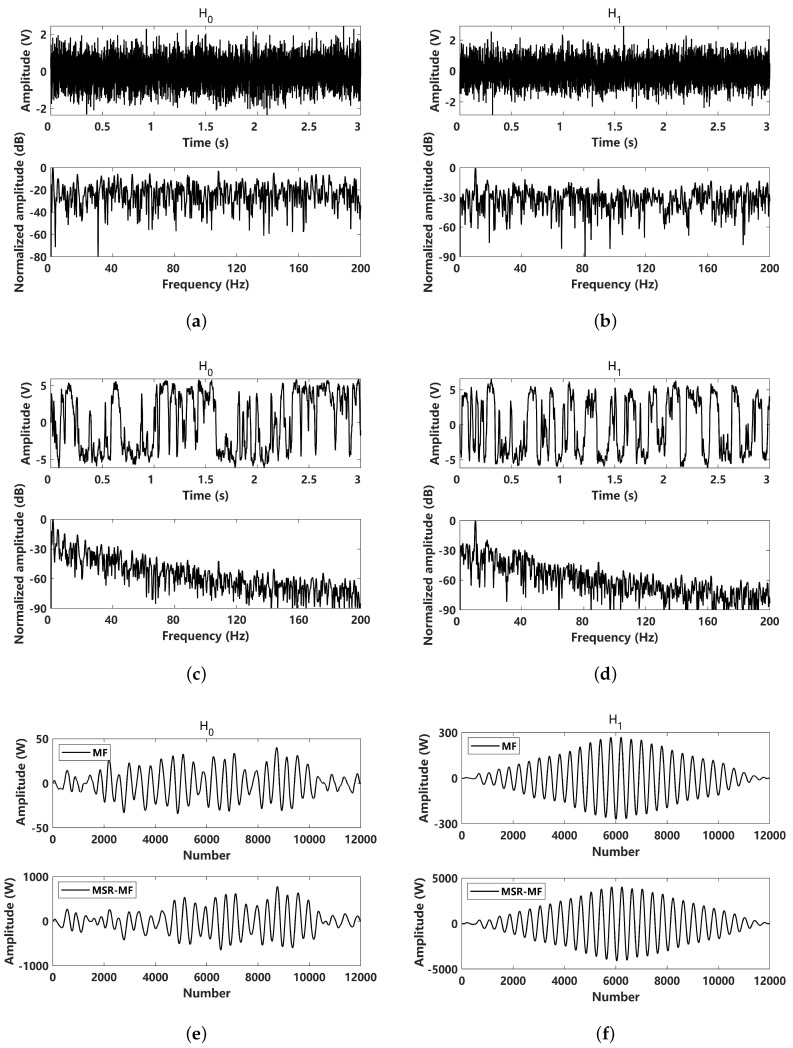
Comparison of input and MSR output results of $H_{0}$ and $H_{1}$ hypothesis under Gaussian noise (α=2): (**a**) received signal under $H_{0}$ hypothesis (A=0); (**b**) received signal under $H_{1}$ hypothesis (A=0.1); (**c**) MSR output signal under $H_{0}$ hypothesis (A=0); (**d**) MSR output signal under $H_{1}$ hypothesis (A=0.1); (**e**) matched filtering processed to the received signal and the corresponding MSR output signal under the $H_{0}$ hypothesis (A=0); (**f**) matched filtering processed to the received signal and the corresponding MSR output signal under the $H_{1}$ hypothesis (A=0.1).

To evaluate the detection performance, a simulation comparison of the received signals and the corresponding MSR outputs under two hypotheses was made, as shown in Figure 5. For $H_{0}$ hypothesis, the received signal is pure noise, and for the $H_{1}$ hypothesis, the received signal is a noisy sinusoidal signal with the amplitude A=0.1. The 10 Hz signal can be clearly identified in the frequency domain. The results obtained by MSR processing results are shown in Figure 5c,d. In view of the normalized power spectrum of the two hypothesis, it can be seen that the MSR output energy is more likely to tend toward a low frequency that follows a Lorentz distribution, as mentioned previously. For the $H_{0}$ hypothesis, the MSR output signal follows the noise response when the peak in power spectrum is close to 0 Hz, while for the MSR output power spectrum of the $H_{1}$ hypothesis, the peak is located at 10 Hz. The MF and MSR-MF of the two hypotheses are shown in Figure 5e,f, where the results of MF and MSR-MF are so close that they cannot exactly predict the detection performance.

**Figure 6:**
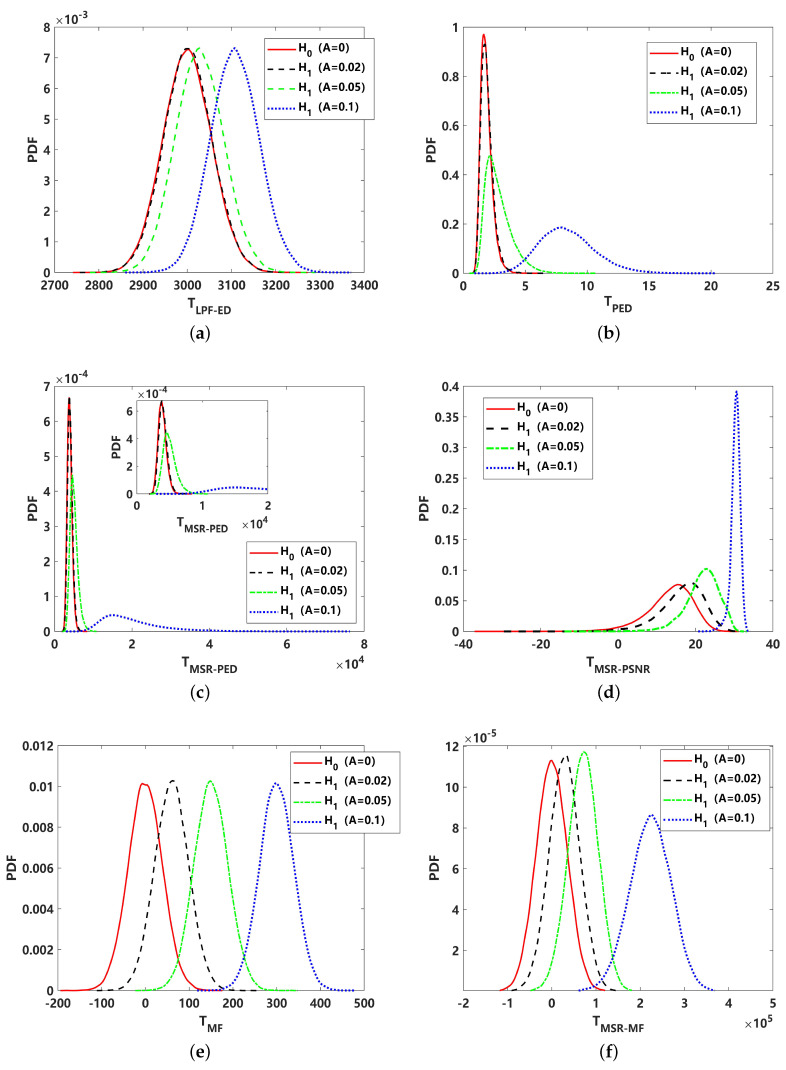
Comparison of probability density function (PDF) of different test statistics under Gaussian noise (α=2): (**a**) energy detector with low pass filter for received signal ($T_{LPF-ED}$); (**b**) PED for received signal ($T_{PED}$); (**c**) PED for MSR output signal ($T_{MSR-PED}$); (**d**) PSNR for MSR output signal ($T_{MSR-PSNR}$); (**e**) matched filtering for received signal ($T_{MF}$); (**f**) matched filtering for MSR output signal ($T_{MSR-MF}$).

**Figure 7:**
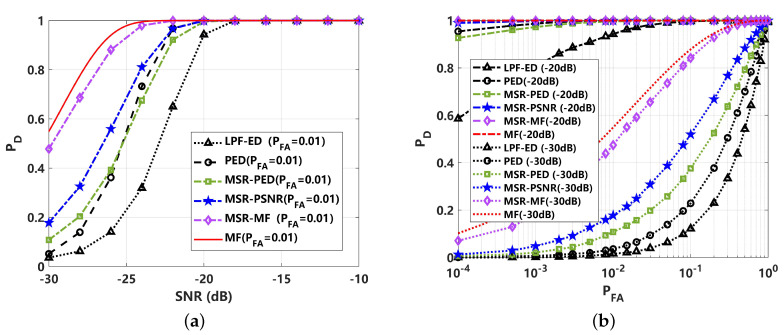
Detection performance comparison of different methods under Gaussian noise (α=2): (**a**) detection probability ($P_{D}$) curve, varying with SNR ($P_{FA}=0.01$); (**b**) receiver operating curve (ROC) corresponding to −20 dB and −30 dB.

To analyze the effect on the detection performance of the different test statistics of detectors, a comparison of the probability density function (PDF) with $10^{5}$ statistics is presented in Figure 6. The test statistics include an energy detector with a low pass filter for received signal $T_{LPF-ED}$, PED for received signal $T_{PED}$, PED for MSR output signal $T_{MSR-PED}$, PSNR for MSR output signal $T_{MSR-PSNR}$, matched filter for received signal $T_{MF}$, and matched filter for MSR output signal $T_{MSR-MF}$. The curve of the $H_{0}$ hypothesis and $H_{1}$ hypothesis with different signal amplitudes can clearly reveal their detection performance, where the $T_{LPF-ED}$ should be inferior in all results. This is further revealed in Figure 7. In comparison with Figure 6b,c, corresponding to $T_{PED}$ and $T_{MSR-PED}$, it can be seen that the performance is close. According to the detection curve in Figure 7, PED is better under a higher SNR, while MSR-PED is superior under lower-SNR conditions. For real applications, the detection probability $P_{D}$ generally requires that $P_{D}\geq 80\%$; hence, the PED may be a better choice than MSR-PED in this instance. The PDF of the proposed $T_{MSR-PSNR}$ is shown in Figure 6d. Intuitively, it can be seen that the $T_{MSR-PSNR}$ is superior to the PDF corresponding to the $H_{0}$, and the $H_{1}$ hypothesis is more likely to be separated. This indicates that utilizing $T_{MSR-PSNR}$ can lead to a better detection performance, and this result is further validated, as shown in Figure 7. This means that the proposed MSR-PSNR is efficient in improving the detection performance, especially under low-SNR conditions. By setting the detection probability at $P_{D}$ = 0.8, the minimum detectable SNR of MSR-PSNR can reach −24 dB. The ROC curves corresponding to −20 dB and −30 dB are given in Figure 7b. It can be seen that MSR-PSNR is superior with a different $P_{FA}$, especially in improving the detection performance under low-false-alarm-rate conditions. For MSR-MF and MF detectors, it can be seen that MF is superior. This indicates that MSR processing cannot break through the constraints of the optimal detection theory under Gaussian noise.

In summary, the detection of weak periodic signals with MSR can essentially be regarded as a special nonlinear filter, of which the performance of a suboptimal detector can improved, especially under low-SNR conditions. Constructing test statistics using peak SNR can achieve a better detection performance, especially under low-SNR and low-false-alarm-rate conditions.

### 4.2. Detection Performance Analysis under Non-Gaussian Impulsive Noise (α=1.5)

**Figure 8:**
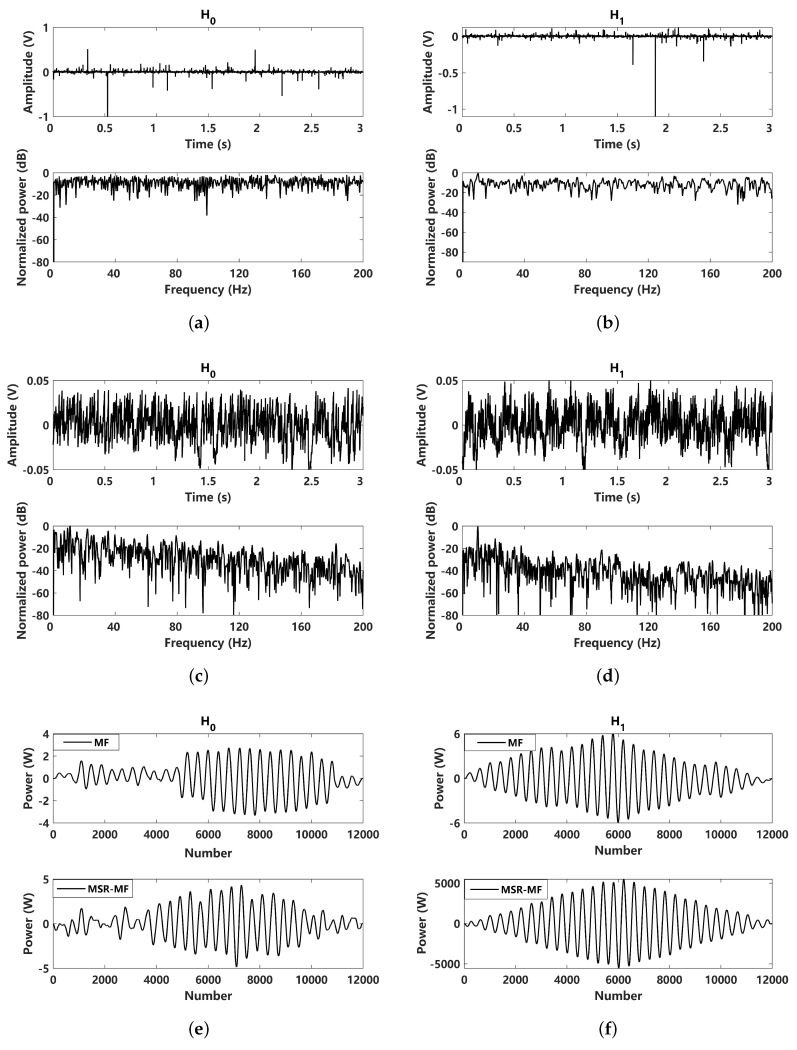
Comparison of input and MSR output results under $H_{0}$ and $H_{1}$ hypotheses (α=1.5): (**a**) received signal under $H_{0}$ hypothesis (A=0); (**b**) received signal under $H_{1}$ hypothesis (A=0.5); (**c**) MSR output signal under $H_{0}$ hypothesis (A=0); (**d**) MSR output signal under $H_{1}$ hypothesis (A=0.5); (**e**) matched filtering processed to the received signal and the corresponding MSR output signal under $H_{0}$ hypothesis (A=0); (**f**) matched filtering processed to the received signal and the corresponding MSR output signal under $H_{1}$ hypothesis (A=0.5).

In the last subsection, the detection performance is evaluated under Gaussian noise. Here, the non-Gaussian impulsive noise conditions with typical α=1.5 is further considered. A simulation comparison of the received signals and the corresponding MSR outputs under two hypothesis is presented in Figure 8. The received signals of the two hypotheses are shown in Figure 8a,b. For impulsive noise, it can be seen that the SNR of the 10 Hz characteristic frequency is greatly affected. Through MSR processing, the corresponding results can be obtained, as shown in Figure 8c,d. The normalized power spectrum of the $H_{0}$ hypothesis follows a Lorentz distribution, while for the MSR output power spectrum of the $H_{1}$ hypothesis, the 10 Hz peak can be clearly identified. This means that the proposed MSR methods can be more effective in dealing with non-Gaussian impulsive noise. The MF and MSR-MF of the two hypotheses are shown in Figure 8e,f, suggesting that the results of the MSR-MF can be improved and a better detection performance can be achieved.

**Figure 9:**
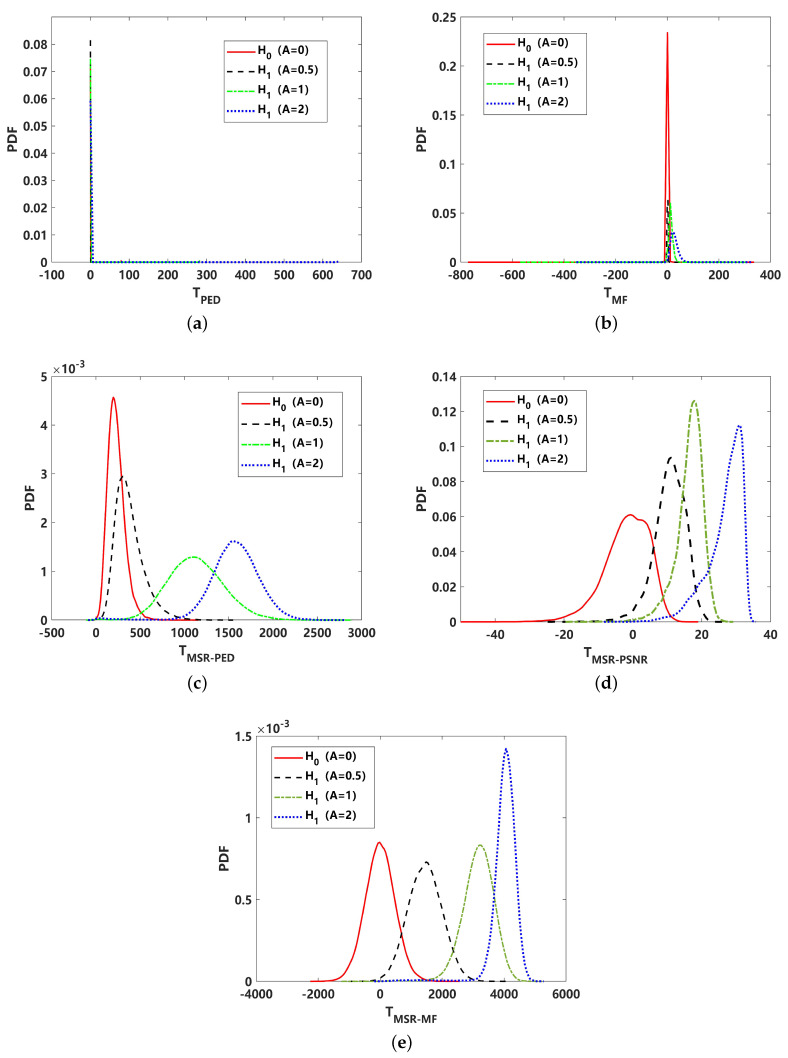
Comparison of probability density function (PDF) of different test statistics under non-Gaussian impulsive noise (α=1.5): (**a**) PED for received signal ($T_{PED}$); (**b**) PED for MSR output signal ($T_{MSR-PED}$); (**c**) PSNR for MSR output signal ($T_{MSR-PSNR}$); (**d**) matched filtering for received signal ($T_{MF}$); (**e**) matched filtering for MSR output signal ($T_{MSR-MF}$).

**Figure 10:**
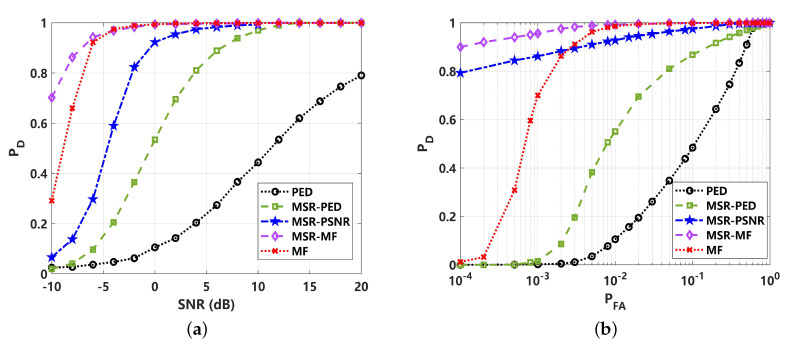
Detection performance comparison of different methods under non-Gaussian impulsive noise (α=1.5): (**a**) detection probability ($P_{D}$) curve, varying with SNR ($P_{FA}=0.01$); (**b**) receiver operating curve (ROC) corresponding to 0 dB.

To better reveal the effect of different test statistics on the detection performance, a comparison of probability density function (PDF) with $10^{5}$ statistics is presented in Figure 9. The test statistics of $T_{PED}$, $T_{MSR-PED}$, $T_{MSR-PSNR}$, $T_{MF}$ and $T_{MSR-MF}$ were adopted. In Figure 9a,b, the PDF is shown to be greatly affected by the heavy tail of impulsive noise. This means that the detection performance of PED and MF would be reduced under low-$P_{FA}$ conditions, which will limit its utilization in passive sonar systems. The PDF of the proposed $T_{MSR-PED}$ and $T_{MSR-PSNR}$ performs better, as illustrated in Figure 9c,d. It can be seen the heavy-tail characteristic of impulsive noise is greatly suppressed. This leads to a better detection performance, as shown in Figure 10. The detection performance is greatly improved by MSR processing, where MSR-PED was detected to be approximately 16 dB lower than PED, at $P_{D}=0.8$. The proposed MSR-PSNR can achieve a better performance, showing an approximately 6 dB improvement compared to MSR-PED, at $P_{D}=0.8$. This indicates that the proposed MSR-PED and MSR-PSNR are superior under non-Gaussian impulsive noise conditions, and the improvement in detection performance is significant. For MSR-MF and MF detectors, it can be seen that MSR-MF is superior. This is because MF is not the optimal detector under non-Gaussian noise conditions. The ROC curves corresponding to 0 dB are given in Figure 10b. It can be seen that the proposed MSR-PSNR is superior with different $P_{FA}$ rates, especially under low-false-alarm-rate conditions.

In summary, the proposed MSR-PED and MSR-PSNR are superior under non-Gaussian impulsive noise conditions (α=1.5), which can lead to a significant improvment in detecting weak signals. The MSR-PSNR performs better, especially under low-false-alarm-rate conditions.

### 4.3. Detection Performance Analysis under Non-Gaussian Impulsive Noise (α=1)

**Figure 11:**
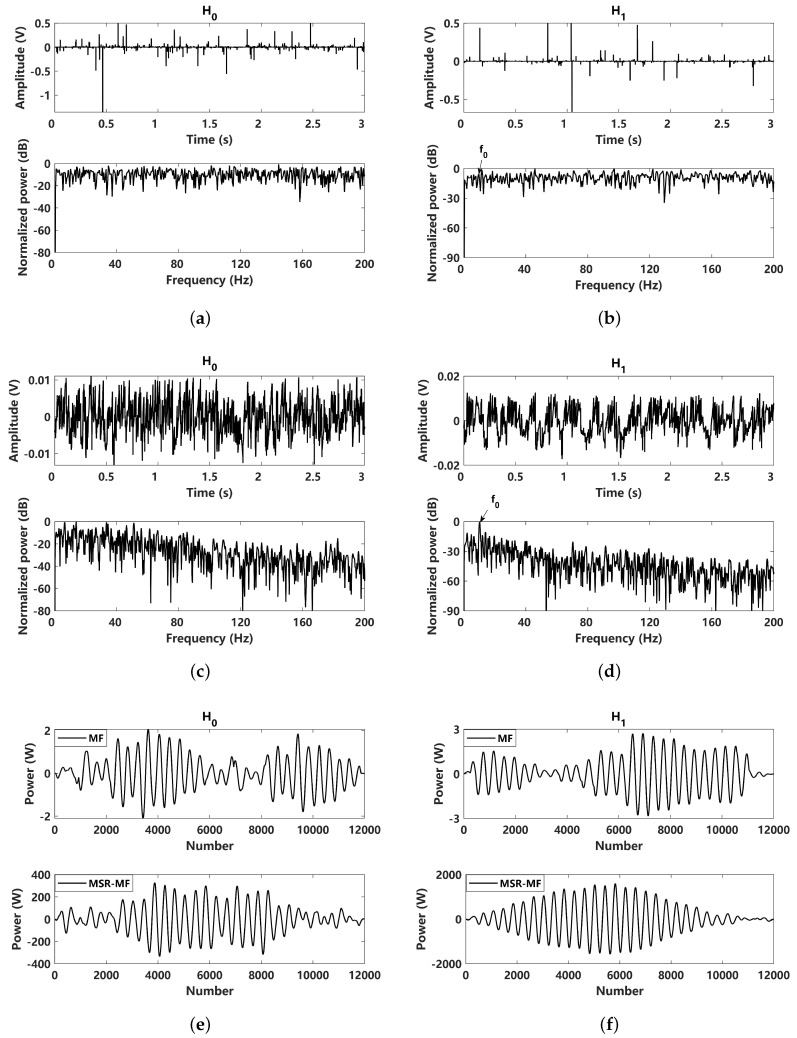
Comparison of input and MSR output results under $H_{0}$ and $H_{1}$ hypotheses (α=1): (**a**) received signal under the $H_{0}$ hypothesis (A=0); (**b**) received signal under the $H_{1}$ hypothesis (A=1); (**c**) MSR output signal under the $H_{0}$ hypothesis (A=0); (**d**) MSR output signal under the $H_{1}$ hypothesis (A=1); (**e**) filtering processes, matched to the received signal and the corresponding MSR output signal under the $H_{0}$ hypothesis (A=0); (**f**) filtering processes, matched to the received signal and the corresponding MSR output signal under the $H_{1}$ hypothesis (A=1).

In the case of strong non-Gaussian impulsive noise, the detection performance of the proposed methods is further evaluated with α=1. With strong non-Gaussian impulsive noise, the received signals are hard to detect. As shown in Figure 11a,b, it is difficult to identify the 10 Hz signal in both the time and frequency domains under the two hypotheses. After MSR processing, the frequency domain results corresponding to the assumption of $H_{0}$ still exhibit typical Lorentz distribution characteristics, where no resonance phenomenon occurs. Corresponding to the assumption of $H_{1}$, comparing the results of Figure 11b,d, a 10 Hz peak is clearly detected in the frequency domain and the local SNR shows a nearly 20 dB improvement. This indicates that the proposed MSR can achieve a superior denoising performance under strong non-Gaussian impulsive noise, and the corresponding MSR-PED and MSR-PSNR are expected to show a significant improvement in their detection performance for passive sonars. In view of the results of MF and MSR-MF, it can be intuitively seen that MSR-MF has significant advantages.

**Figure 12:**
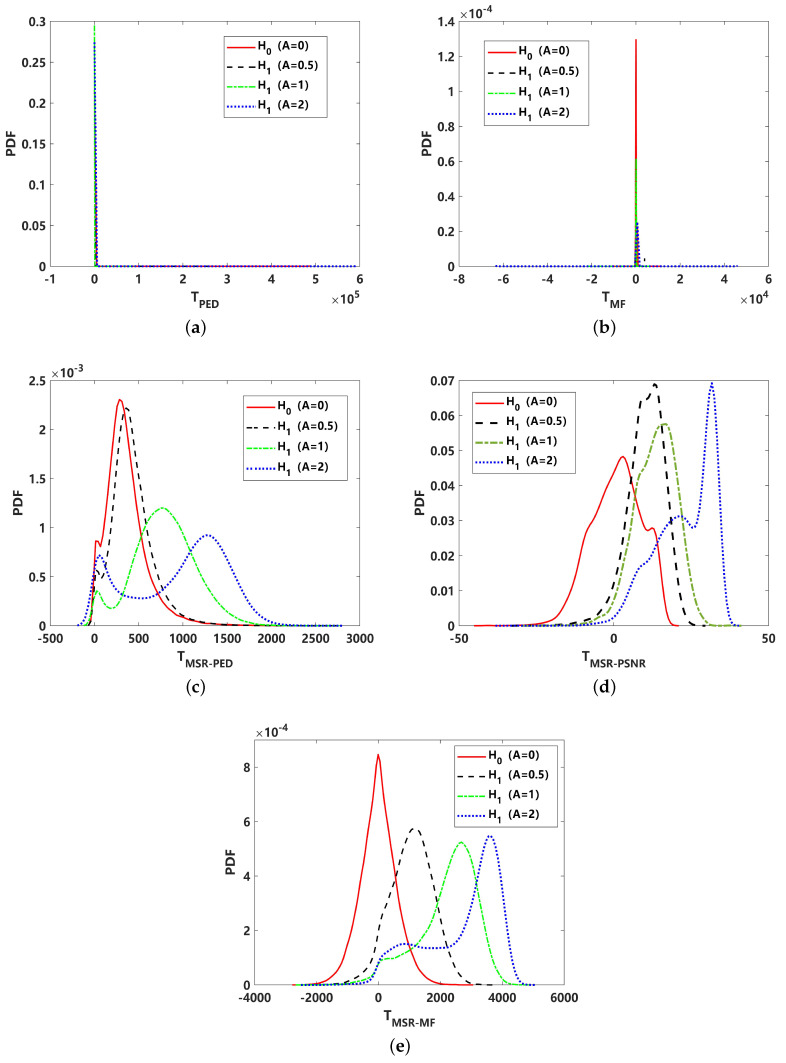
Comparison of probability density function (PDF) of different test statistics under non-Gaussian impulsive noise (α=1): (**a**) PED for received signal ($T_{PED}$); (**b**) PED for MSR output signal ($T_{MSR-PED}$); (**c**) PSNR for MSR output signal ($T_{MSR-PSNR}$); (**d**) matched filtering for received signal ($T_{MF}$); (**e**) matched filtering for MSR output signal ($T_{MSR-MF}$).

**Figure 13:**
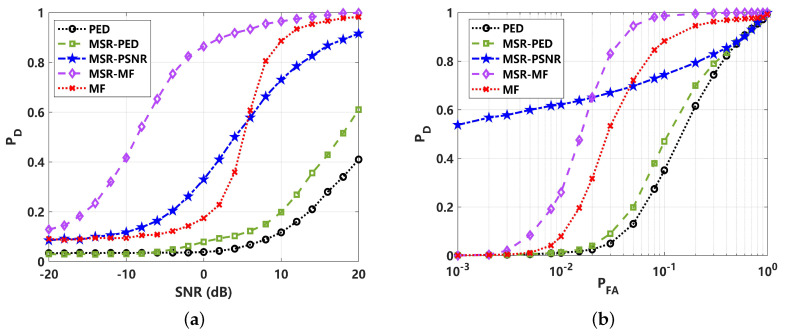
Detection performance comparison under non-Gaussian impulsive noise (α=1): (**a**) detection probability ($P_{D}$) curve with varied SNR ($P_{FA}=0.05$); (**b**) receiver operating curve (ROC) corresponding to 10 dB.

The PDF distributions and detection performance comparisons are shown in Figure 12 and Figure 13. Due to the strong impulsive characteristics of α=1, the test statistics for PED and MF are heavy-tailed, as shown in Figure 12a,b, which would lead to a high false alarm rate. The corresponding receiver operating curve (ROC) in Figure 13b can reveal this limit. After MSR processing, the heavy tail of the corresponding PDF distribution is greatly suppressed. However, for the test statistics of $T_{MSR-PED}$, as given in Figure 12c, the PDF exhibits a typical bimodal structure, making it difficult to achieve the desired detection performance under low-false-alarm-rate conditions. This has a significant effect on the energy-based detectors, as well as $T_{MSR-MF}$, which is shown to have a slight bimodal structure in Figure 12e. For these reasons, the performance of all energy-based detectors decreases under low-false-alarm-rate conditions. These are clearly revealed in the comparison of ROC performance with PED, MF, MSR-PED, and MSR-MF, where the detection performance quickly decreases when $P_{FA}\leq 0.05$, as shown in Figure 13b. This shortcoming can be readily overcome by the proposed MSR-PSNR detector, which can suppress the bimodality of its PDF distribution, as shown in Figure 12d. The detection performance, as shown in Figure 13, further verified that the proposed MSR-PSNR method could improve the detection performance, especially under low-false-alarm-rate conditions ($P_{FA}\leq$ 0.02).

In summary, as the non-Gaussian noise and pulsatility increase, the performance of energy-based detectors will be greatly affected, especially under low-false-alarm-rate conditions. In such cases, the proposed MSR-PSNR method can effectively address this problem. From the perspective of signal processing gains, a significant improvement in local SNR is achieved by MSR processing. The essence of its detection performance gain under low-false-alarm-rate conditions can be understood as a beneficial change in the PDF distribution of the test statistics after special nonlinear transformation. How to better utilize the nonlinear filtering effect to optimize the PDF distribution is still an open problem that is worth future study. From the perspective of its application, MSR’s advantages in detecting low SNR and low false alarm rates in conditions with complex background noise can provide a potential solution for passive sonars in very-low-frequency marine environments with strong impulsive interferences.

## 5. Experimental Verification

**Figure 14:**
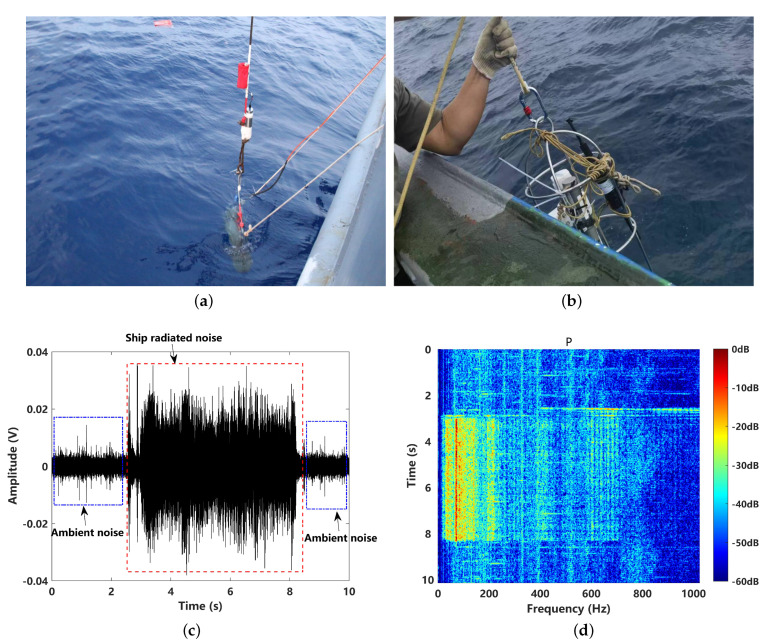
Sea experiment: (**a**) the deployment of low-frequency broadband sound source UW350; (**b**) the receiver with an ocean sonics hydrophone; (**c**) the received signal in the time domain; (**d**) the received signal in the time–frequency domain (received at 500 m distance).

To better reveal the practical application performance, a set of sea experiment data were adopted, collected in the South China Sea. The water depth of this sea area is about 38 m, with a flat sand bottom on the seabed. A low-frequency broadband sound source (UW350) was deployed at 10 m’ depth with a fixed location ($18.251768^{\circ }$ N, $108.905868^{\circ }$ E) to set a prereceived ship-radiated noise fragment. An ocean sonics hydrophone was utilized to receive signals that were deployed at the same depth by a moving ship at different distances. The hydrophone was calibrated with a broadband frequency band ranging from 10 Hz to 200 kHz. The sensitivity of the hydrophone was −178 dB re 1 V/μPa. To better reveal the low-frequency signal, the sampling rate $f_{s}$ was set to 10.24 kHz. A 10 s fragment of the received signal in the time domain and the time–frequency domain are illustrated in Figure 14c,d. A very low-frequency periodic line’s spectral characteristics are shown at 66 Hz, along with its high-order harmonics of the target signal.

**Figure 15:**
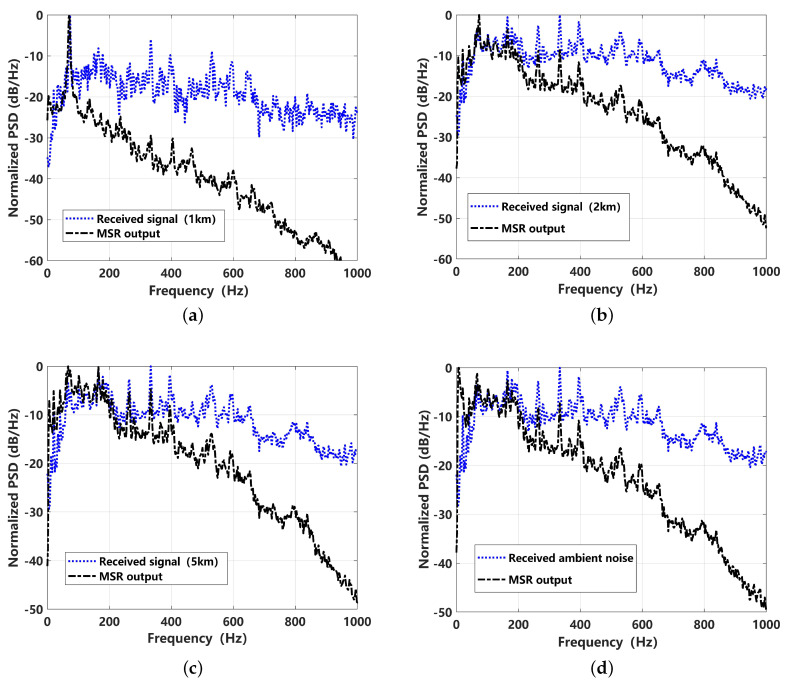
Normalized power spectral density (PSD) comparison of received signal and the corresponding MSR output for different distances: (**a**) 1 km; (**b**) 2 km; (**c**) 5 km; (**d**) ambient noise.

The MSR processing results for the data received at different distances and different levels of ambient noise are shown in Figure 15. It can be seen that the output of MSR has typical low-pass filtering characteristics that follow the Lorentz distribution. As the received distance increases, the resonance is more likely to respond to enhance the lower-frequency noise. For the received signals corresponding to 1 km and 2 km, the resonance phenomenon can occur at the characteristic frequency of 66 Hz, while for the received signal corresponding to 5 km, the MSR output response is close to the ambient noise.

**Figure 16:**
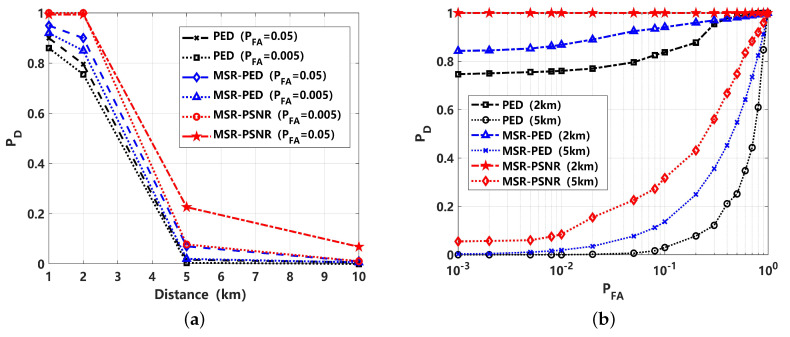
Detection performance comparison for ship-radiated signals: (**a**) detection probability ($P_{D}$) curve, varied with distance; (**b**) receiver operating curve (ROC).

The detection performance of PED and the proposed MSR-PED and MSR-PSNR is compared in Figure 16. It can be seen that the performance of the MSR-PED is better than PED under different false alarm probabilities. This should refer to the denoised performance of MSR. The detection performance of the proposed MSR-PSNR is the best. This is in accordance with the previous simulation analyses. Under low-false-alarm-rate conditions, the proposed MSR-PSNR could also be superior.

## 6. Conclusions

In this paper, a novel matched stochastic resonance (MSR)-based weak signal detection model was established, and two MSR-based detectors, named MSR-PED and MSR-PSNR, were proposed to address the problem of weak signal detection under non-Gaussian impulsive background noise. Comprehensive detection performance analyses in both Gasussian and non-Gasussian impulsive noise conditions were presented. For a typical non-Gasussian impulsive noise assumption with α=1.5, MSR-PED and MSR-PSNR can achieve approximately 16 dB and 22 dB improvements, respectively, with a determined detection performance, compared to the classical PED method. For stronger non-Gaussian impulsive noise conditions corresponding to α=1, the improvement in detection performance is more significant and can be superior to the matched filtering (MF) performance with prior information. The proposed MSR-PSNR methods can resolve the challenging problem of a high false alarm rate caused by heavy-tailed impulsive noise. From the perspective of signal processing, the improvement achieved by MSR-PSNR in the detection performance under low-false-alarm-rate conditions can be understood as a beneficial change in the PDF distribution of the test statistics after special nonlinear transformation. How to better utilize the nonlinear filtering effect to optimize the PDF distribution is still an open problem that is worthy of future study. From the application perspective, the advantages of MSR in detecting low SNR and low false alarm rates with complex background noise can provide a potential solution for passive sonars operating in complex marine environments with strong impulsive interferences. In view of this, this work can make a positive contribution to resolving the challenges of underwater passive sonar detection with non-Gaussian impulsive background noise, and can provide important guidance for future research work. 

## Data Availability

Data sharing is not applicable to this article.
